# Conditioned Extracellular Vesicles Derived from Dedifferentiated Fat Cells Promote Bone Regeneration by Altering MicroRNAs

**DOI:** 10.3390/pharmaceutics16111430

**Published:** 2024-11-10

**Authors:** Yingyi Shen, Zihang Xu, Xinyu Zhang, Zidi Zhai, Yaqin Wu, Fang Qu, Chun Xu

**Affiliations:** 1 Department of Prosthodontics, Shanghai Ninth People’s Hospital, Shanghai Jiao Tong University School of Medicine, Shanghai 200011, China; 2 College of Stomatology, Shanghai Jiao Tong University, Shanghai 200011, China; 3 National Center for Stomatology and National Clinical Research Center for Oral Diseases, Shanghai 200011, China; 4 Shanghai Key Laboratory of Stomatology and Shanghai Research Institute of Stomatology, Shanghai 200011, China; 5 Shanghai Engineering Research Center of Advanced Dental Technology and Materials, Shanghai 200125, China

**Keywords:** dedifferentiated fat cells, extracellular vesicles, bone regeneration, osteogenesis

## Abstract

**Background**: Extracellular vesicles (EVs) derived from stem cells demonstrate significant potential in bone regeneration. Adipose tissue is regarded as a stem cell reservoir with abundant reserves and easy accessibility. Compared to adipose-derived stem cells (ASCs), dedifferentiated fat cells (DFATs) possess similar stem cell characteristics but exhibit greater proliferative capacity, higher homogeneity, and an enhanced osteogenic differentiation potential. This study is the first to examine the effect of DFATs-derived EVs on bone regeneration and elucidate their potential mechanisms of action. **Methods**: Primary DFATs were cultured using the “ceiling culture” method and EVs were isolated by ultracentrifugation and characterized. Experiments were performed to assess the impact of the EVs on the proliferation, migration, and osteogenesis of bone marrow mesenchymal stem cells (BMSCs). Subsequently, high-throughput miRNA sequencing was conducted on the EVs derived from DFATs that had undergone 0 days (0d-EVs) and 14 days (14d-EVs) of osteogenic differentiation. **Results**: The results indicated that the EVs derived from DFATs which experienced 14 days of osteogenic induction significantly promoted the proliferation, migration, and osteogenic differentiation of BMSCs. High-throughput sequencing results revealed that up-regulated miRNAs in the 14d-EVs were primarily involved in biological processes such as the Notch signaling pathway and the positive regulation of cell movement and migration. The target genes of these differently expressed miRNAs were enriched in osteogenesis-related signaling pathways. **Conclusion**: This study innovatively demonstrated that conditioned EVs (14d-EVs) derived from DFATs promoted the osteogenic differentiation of BMSCs via miRNAs, offering a promising cell-free therapeutic option for bone defect.

## 1. Introduction

Oral and maxillofacial bone defects impact facial aesthetics and lead to functional impairments. Stem cell-derived extracellular vesicles (EVs) have emerged as a prominent research focus in the field of bone regeneration [1,2,3]. Compared to cell therapy, EVs offer advantages such as reduced immunogenicity, lower production costs, enhanced stability, simpler sterilization processes, and more convenient storage conditions [4,5,6]. These characteristics make EVs-based cell-free therapies a promising alternative to traditional stem cell therapies.

The biological function of EVs largely depends on the source and physiological status of the donor cells [7]. Therefore, identifying suitable source cells is crucial for enhancing the curative efficacy of EVs. Compared to bone marrow mesenchymal stem cells (BMSCs), the extraction of stem cell-derived EVs from adipose tissue offers several advantages. Firstly, adipose tissue is widely distributed and readily accessible. The number of stem cells obtained from adipose tissue is far greater than that obtained from the same amount of bone marrow [8]. Furthermore, adipose-derived stem cells (ASCs) exhibit greater proliferative capacity during in vitro culture and expansion [9], ensuring a steady supply of EVs for therapeutic applications. Consequently, adipose tissue-derived stem cells are anticipated to improve the yield and biological effects of EVs, which holds significant scientific importance.

Recently, the role of dedifferentiated fat cells (DFATs) in bone regeneration has garnered increasing attention [10,11,12,13]. Adipogenic differentiation was previously deemed an irreversible terminal process, until 1986, when Sugihara et al. [14] first successfully cultured DFATs using the “ceiling culture” method. Subsequent studies have confirmed that DFATs express stem cell-related markers [15] and possess the potential to differentiate into various cell types, including adipocytes, osteoblasts, chondrocytes, and neurocytes [11,16,17,18,19]. Compared to ASCs, DFATs exhibit superior proliferation capabilities, greater homogeneity, and an enhanced osteogenic differentiation potential [10,11]. These characteristics suggest that DFATs may offer significant advantages as donor cells for EVs in bone regeneration.

EVs regulate the repair and regeneration of injured sites by influencing the proliferation, migration, differentiation, and immune environment of recipient cells. Among these processes, miRNAs may play a crucial role in mediating their biological effects [20,21]. Studies have shown that stem cell-derived EVs induced through osteogenic differentiation exhibit a more pronounced osteogenic effect [22,23]. The variability in osteogenic effects may be attributed to differences in miRNAs between EVs derived from stem cells which have experienced osteogenic versus non-osteogenic induction processes.

Currently, research on DFATs primarily focuses on cell therapy, with few studies investigating the impact of DFATs-derived EVs on tissue regeneration. This study is the first to explore the role and mechanisms of DFATs-derived EVs in enhancing bone regeneration via miRNAs, aiming to provide a reference for the treatment of oral and craniomaxillofacial bone defects.

## 2. Materials and Methods

### 2.1. Primary Culture of DFATs and BMSCs

All animal experiments were performed in compliance with the ARRIVE guidelines and the National Research Council’s Guide for the Care and Use of Laboratory Animals. The experimental protocol was approved by the ethics committee of Shanghai Ninth People’s Hospital, Shanghai Jiao Tong University School of Medicine, with the ethical approval number SH9H-2024-A1026-1. DFATs were isolated from the subcutaneous inguinal tissue of 10-week-old female SD rats according to the protocol reported in a previous report [11]. In brief, the abdominal adipose tissue was cut as much as possible and digested with 0.1% (*w*/*v*) type I collagenase (BioFroxx, Anprotec, Germany) in a container at 37 °C for 1 h. The digested products were centrifuged at 135× *g* for 3 min. The floating layer at the top that contained the mature adipocytes was collected and placed in a T25 culture flask full of Dulbecco’s modified eagle medium (DMEM) (Gibco, Grand Island, NY, USA) supplemented with 20% fetal bovine serum (FBS) (ExCell Bio, Suzhou, China) and 1% penicillin-streptomycin (Gibco, Grand Island, NY, USA). The culture flask was inverted and placed in a humidified incubator with 5% CO_2_ at 37 °C. After 7 days, the flask was turned upside down so that DFATs were cultured as adherent cells.

Femur bone marrow from 3-week-old male SD rats was utilized for primary BMSCs isolation according to a previously reported protocol [24]. All the media were refreshed every 3 days and cells at third passage were used in the following experiments.

### 2.2. Osteogenic, Adipogenic and Chondrogenic Differentiation of DFATs

Third-passage DFATs in the logarithmic growth phase were seeded into six-well plates. When the cell density reached 60~80%, osteogenic (Cyagen, Santa Clara, CA, USA), adipogenic (Cyagen, Santa Clara, CA, USA), and chondrogenic (Cyagen, Santa Clara, CA, USA) differentiation media were applied for subsequent culturing. After 14~21 days of differentiation induction, the cells were fixed with 4% neutral formaldehyde solution (Biosharp, Anhui, China) for 30 min. Alizarin red, oil red O, and Alcian blue dyes were then used for staining. The staining was observed under a microscope and images were captured.

### 2.3. Identification of DFATs

DFATs were resuspended in flow cytometry buffer, and the cell concentration was adjusted to 3 × 10^6^ cells/mL. A total of 100 μL of the cell suspension was incubated with 2 μL of CD11b-FITC, CD29-FITC, CD34-FITC, CD45-FITC, CD73-FITC, and CD90-FITC antibodies (Cyagen, Santa Clara, CA, USA) at 4 °C for 30 min. The samples were then washed and resuspended with PBS. The fluorescence intensity of DFATs-labeled antibodies was immediately detected using a flow cytometer (BD Biosciences, San Jose, CA, USA), and the results were analyzed using FlowJo V10.10.

### 2.4. Isolation and Characterization of EVs

When the third-passage DFATs reached 80% confluence, the regular culture medium was replaced with DMEM supplemented with 10% EVs-free fetal bovine serum (Sigma-Aldrich, St. Louis, MO, USA) and 1% penicillin–streptomycin. After 48 h of culturing, the cell supernatant was collected, and DFATs-derived EVs were isolated by ultracentrifugation. Specifically, the supernatant was centrifuged at 4 °C by freezing centrifuge (Thermo Fisher, Waltham, MA, USA) under the following conditions: it was centrifuged at 300× *g* for 10 min, followed by 2000× *g* for 10 min and 10,000× *g* for 30 min. Subsequently, the supernatant underwent ultracentrifugation for 70 min at 110,000× *g* at 4 °C by ultra-centrifuge (Beckman Coulter, Brea, CA, USA). According to the literature [25], all the EVs used in this study were stored at −80 °C within 4 weeks after extraction to maintain the stability and particle diameter. The collected EVs were resuspended in PBS and stored at −80 °C. The morphology of the EVs was observed using a transmission electron microscope (TEM) (Hitachi, Tokyo, Japan). The particle size and concentration were measured using a nanoparticle size analyzer (NanoSight, Malvern, UK). Additionally, Western blot analysis was performed to detect surface marker proteins using specific antibodies for CD81 (1:1000, Abclonal, Woburn, MA, USA), TSG101 (1:1000, Affinity, Nottingham, UK), and Calnexin (1:2000, Abcam, Cambridge, UK).

### 2.5. Internalization Assay of EVs

BMSCs were plated at a density of 1.5 × 10^5^ cells/mL in a 35 mm confocal culture dish. EVs were diluted to a concentration of 100 μg/mL using 500 μL of Dilute C (Sigma-Aldrich, St. Louis, MO, USA). The dye working solution was prepared by adding 4 μL of PKH-67 dye (Sigma-Aldrich, St. Louis, MO, USA) to 500 μL of Dilute C. It was then combined with the diluted EVs suspension and incubated in the dark at room temperature for 4 min. To quench excess dye, 2 mL of 0.5% FBS was added. Finally, the prepared mixture was transferred to a confocal culture dish so that the PKH-67-labeled EVs were incubated with the BMSCs for 12 h. Following co-culturing, the cytoskeleton and nuclei were subsequently stained with iFluor555 phalloidin (YEASEN, Shanghai, China) and DAPI (YEASEN, Shanghai, China), respectively. The internalization of the EVs by the BMSCs was observed using a laser confocal microscope (Leica, Wetzlar, Germany).

### 2.6. Determination of Optimal Conditioned EVs

To determine the optimal duration of osteogenic induction and the concentration of DFATs-derived EVs, EVs were isolated from the cell supernatant at 0, 3, 7, and 14 days of osteogenic induction, which were designated as 0d-EVs, 3d-EVs, 7d-EVs, and 14d-EVs, respectively. The concentration of EVs was measured using a BCA protein assay kit (Beyotime, Shanghai, China). Second-generation BMSCs in the logarithmic growth phase were seeded into a 12-well plate at a density of 2 × 10^5^ cells per well. Once the cells adhered, the proliferation medium was replaced with 1.5 mL of osteogenic induction medium containing varying concentrations of EVs. To evaluate the osteogenic effect of DFATs-derived EVs on BMSCs, BMSCs were exposed to different groups of EVs. After 7 days of osteogenic induction, alkaline phosphatase (ALP) staining was performed using the BCIP/NBT alkaline phosphatase colorimetric kit (Beyotime, Shanghai, China). ALP activity was assessed using an alkaline phosphatase detection kit (Beyotime, Shanghai, China) according to the manufacturer’s instructions.

### 2.7. Proliferation Assay

According to the results from Section 2.6, 10 μg/mL of EVs was identified as the optimal concentration for subsequent experiments in vitro. The 96-well plates were inoculated with BMSCs, and 100 μL of a culture medium containing 0d-EVs, 14d-EVs, or PBS was added after the cells were adhered to the wall. On the first, second, and third day, 10 μL of CCK-8 solution (Dojindo, Kumamoto, Japan) was added to each well. After a 30 min incubation at 37 °C, absorbance at 450 nm was measured using a microplate reader (Biotek, Winooski, VT, USA).

### 2.8. Migration Assay

A transwell culture chamber consists of an 8 μm microporous membrane that separates the upper chamber (6.5 mm in diameter) from the lower chamber (9 mm in diameter). When cells in the upper chamber migrate through the membrane pores into the lower chamber, the migration can be observed microscopically. A total of 600 μL of medium containing 0d-EVs, 14d-EVs, or PBS was added to the lower chamber of a 24-well transwell plate (Corning, Corning, NY, USA), and a BMSCs suspension was added to the upper chamber at a concentration of 1 × 10^5^ cells/mL. The cells were incubated at 37 °C with 5% CO_2_ for 48 h and then fixed with 4% paraformaldehyde and stained with crystal violet (Beyotime, Shanghai, China) for 20 min. Non-migrated cells in the upper chamber were gently removed with a cotton swab. Cell migration observation and quantitative analysis were then conducted.

### 2.9. Quantitative Real-Time Polymerase Chain Reaction (qRT–PCR)

BMSCs were seeded into 6-well culture plates. Upon reaching 80% confluence, the complete medium was replaced with osteogenic induction medium, which contained 10%FBS, 1% penicillin–streptomycin, 10 mmol/L sodium β-glycerophosphate, 0.1 μmol/L dexamethasone, and 50 mg/L vitamin C. The cells were divided into three groups based on the EVs content in the induction medium: the PBS group (control) with no EVs, the 0d-EVs group containing 10 μg/mL 0d-EVs, and the 14d-EVs group containing 10 μg/mL 14d-EVs. The medium was refreshed every 3 days, and total RNA was extracted after 7 days of osteogenic induction. The total RNA was extracted using Trizol reagent (Takara, Tokyo, Japan) and reverse transcribed into cDNA with the PrimeScript™ RT kit (Takara, Tokyo, Japan), following the manufacturer’s instructions. Quantitative real-time polymerase chain reaction (qRT–PCR) was conducted using SYBR reagent (YEASEN, Shanghai, China) with GAPDH as an internal control to assess the mRNA expression of osteogenic-related target genes. Quantitative analysis was performed using the 2^−ΔΔCt^ method.

### 2.10. Western Blotting

After 7 days of osteogenic induction in 6-well plates, total proteins were extracted from BMSCs using RIPA lysis buffer (Merck, Kenilworth, NJ, USA) and quantified using a BCA protein assay. Following protein denaturation, sodium dodecyl sulfate-polyacrylamide gel electrophoresis (SDS-PAGE) was performed, and proteins were transferred to polyvinylidene fluoride (PVDF) membranes. The membranes were then blocked with blocking solution (Beyotime, Shanghai, China) and incubated overnight at 4 °C with primary antibodies. Afterward, they were incubated for 1 h with secondary antibodies (YEASEN, Shanghai, China) and developed using enhanced chemiluminescence reagents (Thermo Fisher Scientific, Massachusetts, USA). The intensity of each band was analyzed semi-quantitatively using ImageJ. The antibodies used in this study were as follows: RUNX2 (1:1000, Affinity, Nottingham, UK); BMP2 (1:1000, Affinity, Nottingham, UK); β-actin (1:3000, Abways, Shanghai, China); and horseradish peroxidase-conjugated donkey anti-rabbit IgG (H+L) (1:5000, YEASEN, Shanghai, China).

### 2.11. miRNA Sequencing and Screening

The extraction, library preparation, and sequencing of miRNAs on the 14d-EVs and 0d-EVs were provided by CloudSeq Inc. (Shanghai, China). Differentially expressed miRNAs were filtered by calculating *p*-values and the fold change (FC) between the two groups. FC ≥ 1.0 and *p* < 0.05 were used as thresholds for identifying differential miRNAs. The top 10 target genes of these differentially expressed miRNAs were then analyzed using gene ontology (GO) analysis and the Kyoto Encyclopedia of Genes and Genomes (KEGG) pathway analysis. Graphics of a bioinformatic analysis were created using OmicStudio tools at https://www.omicstudio.cn/tool (accessed on 26 July 2024).

### 2.12. Statistical Analysis

Statistical analysis was performed by SPSS 25.0. Data were presented as mean ± standard deviation. Comparisons between two groups were made using the *t*-test, while comparisons among three groups were conducted using one-way analysis of variance (ANOVA). Tukey’s honest significant difference (HSD) test was employed to assess the significance of differences among groups in ANOVA. Statistical significance was determined based on a two-tailed α level of 0.05, with ****p < 0.0001, *** *p* < 0.001, ** *p* < 0.01, and * *p* < 0.05 indicating significance and ns indicating no significance.

## 3. Results

### 3.1. Identification of DFAT Cells

**Figure 1:**
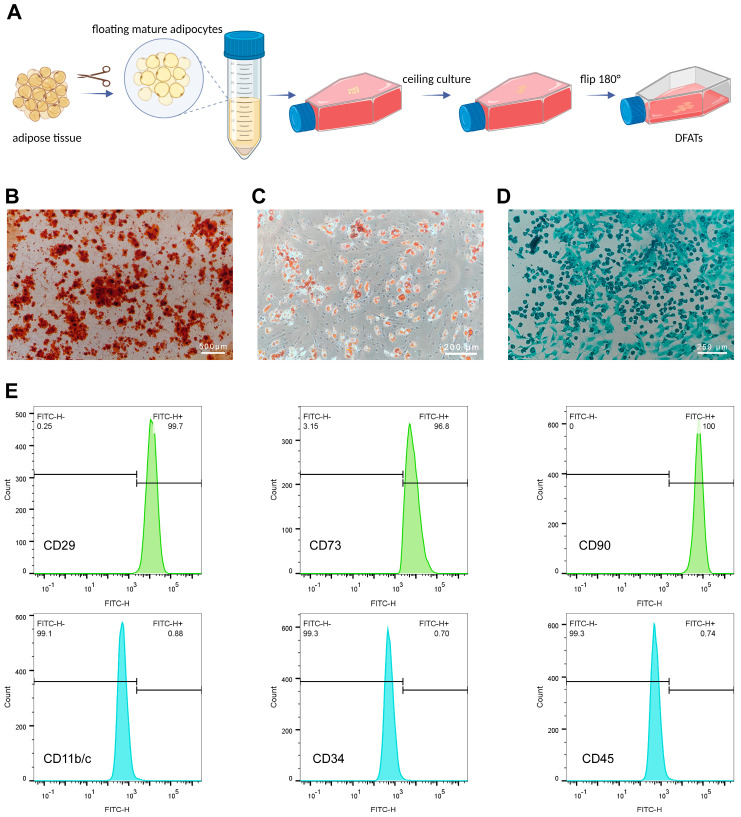
Culture and characterization of DFATs. (**A**) Schematic diagram showing the culture of primary DFATs by “ceiling culture” method (created with BioRender.com). (**B**) ARS staining indicated that DFATs showed osteogenic differentiation potential, with mineralized nodules appearing orange–red. (**C**) Oil red O staining demonstrated that DFATs were capable of differentiating into adipocytes, with lipid droplets appearing red. (**D**) Alcian blue staining revealed that DFATs had chondrogenic differentiation potential, with the acidic mucopolysaccharides appearing blue–green. (**E**) Flow cytometry analysis of DFATs surface markers showed positive expression of mesenchymal stem cell markers CD29 (99.7%), CD73 (96.8%), and CD90 (100%). (DFATs: dedifferentiated fat cells. ARS staining: alizarin red S staining).

Alizarin red S (ARS) staining demonstrated that after 21 days of osteogenic induction and differentiation, DFATs formed mineralized nodules with orange–red calcium salt deposits (Figure 1B), which demonstrates the osteogenic potential of DFATs. Oil red O staining revealed the formation of lipid droplets in DFATs following 21 days of adipogenic induction, which appeared red upon dye binding (Figure 1C). This result demonstrates the adipogenic potential of DFATs. Alcian blue staining indicated that DFATs can differentiate into chondrocytes after 21 days of chondrogenic induction, with the acidic mucopolysaccharides being stained blue–green (Figure 1D).

Flow cytometry was performed to assess the expression of surface markers on the DFATs. Quantitative analysis revealed that mesenchymal stem cell markers such as CD29 (99.7%), CD73 (96.8%), and CD90 (100%) were positive. In contrast, CD11b/c (0.88%), CD34 (0.70%), and CD45 (0.74%) were negative (Figure 1E). These findings confirmed that the DFATs possessed multidirectional differentiation capabilities and expressed stem cell surface markers, indicating their stem cell characteristics.

### 3.2. Characterization of EVs and Their Internalization by BMSCs

**Figure 2:**
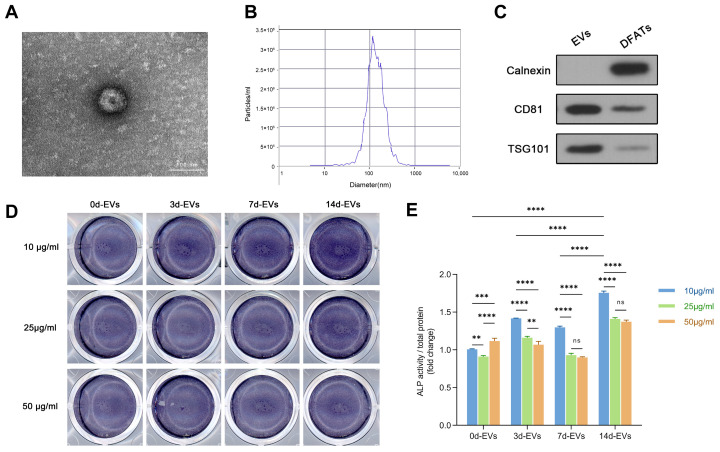
Identification of EVs and determination of their optimal concentration and osteogenic induction duration. (**A**) TEM revealed that EVs possessed a characteristic disk-shaped and double-layer membrane structure. (**B**) NTA demonstrated that diameter of EVs ranged from 79.5 nm to 219.1 nm. (**C**) Western blot analysis indicated that EVs expressed two positive surface markers (CD81 and TSG101) and one negative marker (calnexin). (**D**) ALP staining revealed that DFATs-derived EVs at different concentrations and various osteogenic induction days exhibited different abilities on promoting osteogenesis in BMSCs. (**E**) ALP activity detection indicated that 14d-EVs exhibited highest osteogenic ability at concentration of 10 μg/mL. (EVs: extracellular vesicles. TEM: transmission electron microscopy. NTA: nanoparticle tracking analysis. ALP: alkaline phosphatase. 14d-EVs: EVs derived from DFATs that had undergone 14 days of osteogenic differentiation. ** *p* < 0.01, *** *p* < 0.001, **** *p* < 0.0001, ns: no statistical significance).

TEM revealed that the EVs exhibited a typical disk-shaped, double-layer membrane structure (Figure 2A). Nanoparticle tracking analysis (NTA) indicated that their diameter ranged from 79.5 nm to 219.1 nm, with a peak size of approximately 118.8 nm, which was consistent with the diameter range of EVs (Figure 2B). WB demonstrated that the EVs were positive for the CD81 and TSG101, whereas the endoplasmic reticulum marker calnexin was absent in the EVs but present in the DFATs lysates (Figure 2C).

**Figure 3:**
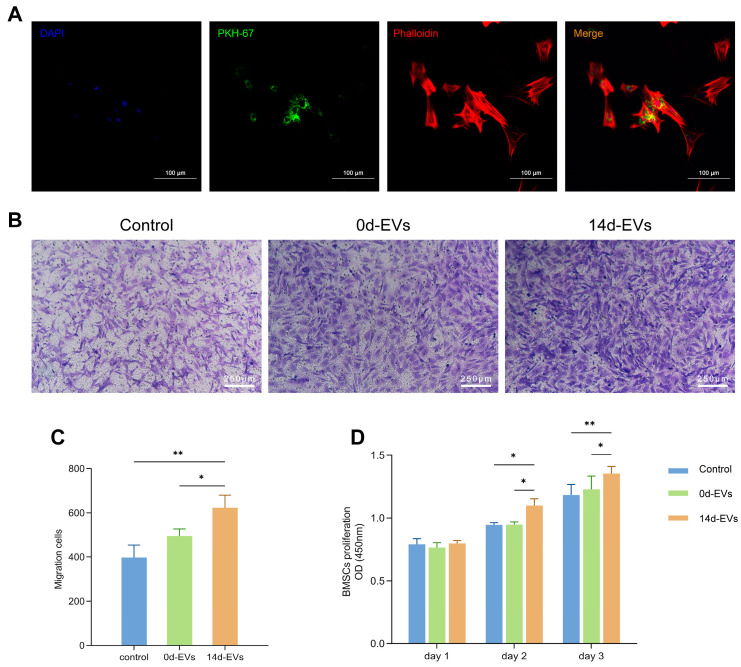
Conditioned EVs enhanced the proliferation and migration of BMSCs. (**A**) The internalization of EVs by BMSCs. iFluor555 labeled the cytoskeleton (red), DAPI labeled the nucleus (blue), and PKH-67 labeled the EVs (green). (**B**) Representative images of a transwell migration assay demonstrated that the EVs enhanced cell migration, with 14d-EVs exhibiting a more pronounced effect compared to the other groups. (**C**) Quantitative analysis of cell migration in the transwell assay revealed that 14d-EVs promoted cell migration more effectively than the other groups. (**D**) The CCK-8 assay indicated that 14d-EVs significantly enhanced the proliferation of BMSCs (* *p* < 0.05, ** *p* < 0.01).

After EVs and BMSCs were co-cultured for 12 h, PKH-67-labeled EVs were observed to aggregate around the nuclei of the BMSCs under a laser confocal microscope (Figure 3A). These results demonstrated that EVs secreted by DFATs during in vitro culture could be successfully extracted and were effectively internalized by the BMSCs.

### 3.3. Screening of the Optimal EVs for Osteo-Inductive Property

The results of ALP staining (Figure 2D) showed that EVs derived from DFATs at different concentrations (10, 25, 50 μg/mL) and various osteogenic induction days (0, 3, 7, and 14 days) exhibited different abilities in promoting osteogenesis in BMSCs. Combined with the quantitative detection of ALP activity (Figure 2E), it was observed that EVs derived from 14 days of osteogenic induction at a concentration of 10 μg/mL resulted in the significantly highest ALP activity compared to the other groups. Therefore, 10 μg/mL was chosen as the optimal concentration for subsequent in vitro experiments.

### 3.4. Conditioned EVs Promoted the Proliferation and Migration of BMSCs

To evaluate the effect of EVs on BMSCs in vitro, the samples were divided into three groups: the control group (containing PBS), the 0d-EVs group (containing 10 μg/mL 0d-EVs), and the 14d-EVs group (containing 10 μg/mL 14d-EVs). The results of the CCK-8 assay (Figure 3D) show that there was no statistical difference among the three groups on the first day. However, on the second and third days, the OD value of the 14d-EVs group was significantly higher than that of the other two groups, indicating a superior ability to promote cell proliferation.

The transwell assay (Figure 3B,C) showed that the cell migration ability after co-culturing with EVs for 48 h was greater than that of the control group. The number of migrating cells in the 14d-EVs group was significantly higher than that in the 0d-EVs group. These results further demonstrate that osteogenic induction pretreatment enhanced the ability of DFATs-derived EVs to promote cell proliferation and migration.

### 3.5. Conditioned EVs Promoted the Osteogenic Differentiation of BMSCs

**Figure 4:**
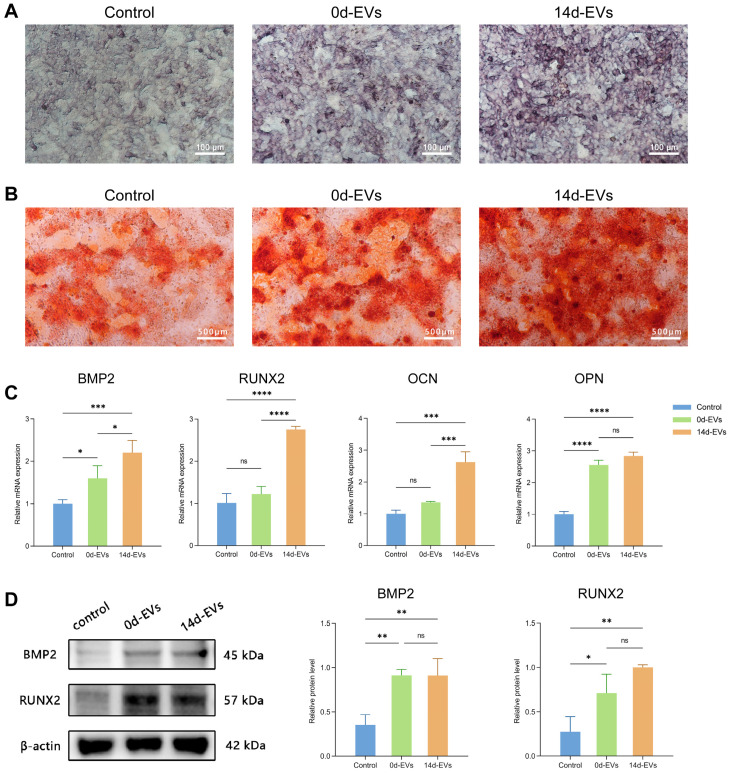
Conditioned EVs promoted osteogenic differentiation of BMSCs. (**A**) Representative images of ALP staining demonstrated that 14d-EVs promoted osteogenic differentiation of BMSCs. (**B**) Representative images of ARS staining indicated that 14d-EVs facilitated formation of mineralized nodules in BMSCs. (**C**) qRT-PCR analysis revealed that 14d-EVs up-regulated mRNA expression levels of BMP2, RUNX2, OCN, and OPN. (**D**) WB analysis showed that 14d-EVs enhanced protein expression of BMP2 and RUNX2. (ALP: alkaline phosphatase. ARS staining: alizarin red S staining. 14d-EVs: EVs derived from DFATs that had undergone 14 days of osteogenic differentiation. * *p* < 0.05, ** *p* < 0.01, *** *p* < 0.001, **** *p* < 0.0001, ns: no statistical significance).

To examine the effect of DFATs-derived EVs on BMSCs osteogenesis, ALP staining, ARS staining, qRT-PCR, and WB were performed. ALP staining microscopic images (Figure 4A) showed that the EVs promoted ALP expression in the BMSCs, with the 14d-EVs group exhibiting a stronger promoting effect compared to the 0d-EVs group. Similarly, the ARS staining (Figure 4B) revealed that the 14d-EVs group formed more mineralized nodules than the other two groups. The qRT-PCR results (Figure 4C) confirmed that the 14d-EVs significantly up-regulated the mRNA expression level of BMP2, RUNX2, OCN, and OPN. The WB analysis (Figure 4D,E) indicated that both the 0d-EVs and 14d-EVs significantly promoted the expression of the osteogenesis-related proteins BMP2 and RUNX2 in the BMSCs.

### 3.6. MiRNA Profiles of EVs Derived from Osteogenically Differentiated DFATs and Undifferentiated DFATs Were Altered

**Figure 5:**
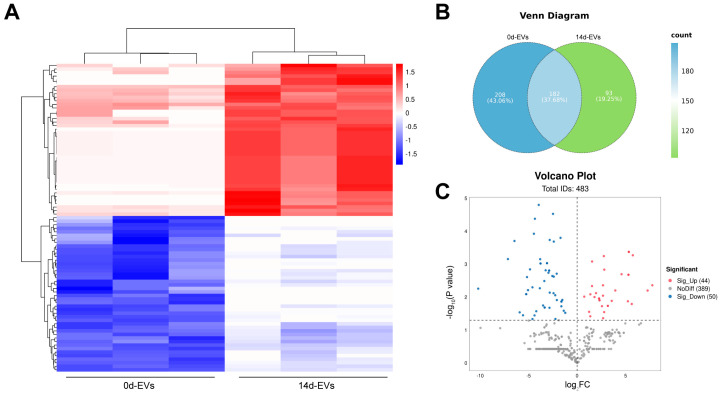
Different miRNA expression profiles in undifferentiated and osteogenic-differentiated DFATs-derived EVs. (**A**) Heat maps from miRNA cluster analysis revealed significant differences between 14d-EVs and 0d-EVs groups, with high consistency within each group. (**B**) Venn diagram illustrated that 93 miRNAs were specific to 14d-EVs group, while 208 miRNAs were specific to 0d-EVs group. (**C**) Volcano plot demonstrated that 44 miRNAs were significantly up-regulated and 50 miRNAs were significantly down-regulated in DFATs-derived EVs after 14 days of osteogenic induction.

High-throughput sequencing technology was used to detect the expression differences in miRNAs in DFATs-derived EVs on days 0 and 14 of osteogenic induction, followed by cluster analysis of these differentially expressed miRNAs. The heat map (Figure 5A) demonstrates high consistency within each group. The Venn diagram (Figure 5B) reveals that a total of 483 miRNAs were identified, with 93 specific miRNAs in the 14d-EVs group and 208 specific miRNAs in the 0d-EVs group. The volcano plot (Figure 5C) indicates that, compared to the 0d-EVs, 44 miRNAs were significantly up-regulated and 50 miRNAs were significantly down-regulated in the 14d-EVs group.

### 3.7. Conditioned EVs Promoted Osteogenic Differentiation Through miRNAs Profiles

**Figure 6:**
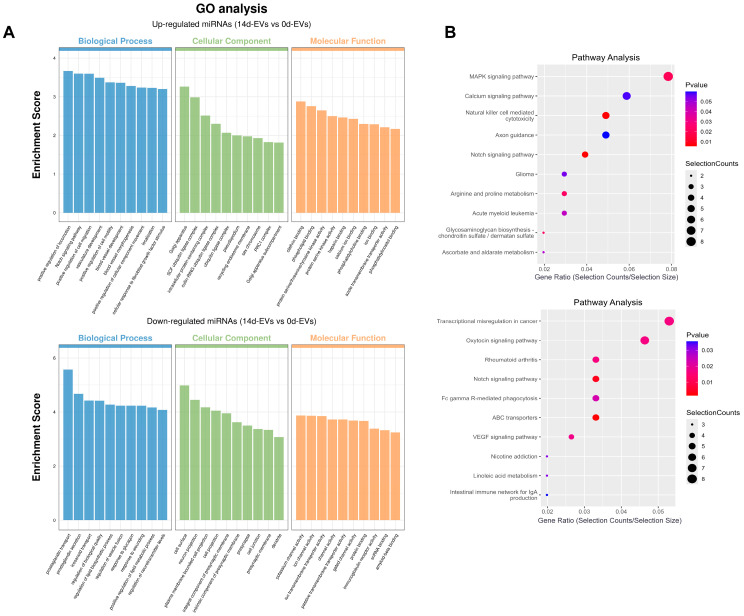
GO and KEGG enrichment analysis on the target genes of differently expressed miRNAs. (**A**) The GO analysis indicated that up-regulated miRNAs in the 14d-EVs group were primarily associated with biological processes like the Notch signaling pathway and the positive regulation of cell motility and movement. (**B**) The KEGG pathway bubble plot illustrates that the target genes of differentially expressed miRNAs were predominantly enriched in pathways related to osteogenic differentiation. (GO: gene ontology. KEGG: Kyoto Encyclopedia of Genes and Genomes).

GO and KEGG enrichment analyses were performed on the differentially expressed miRNA target genes. Bar graphs and bubble graphs were generated for the top ten target genes. The GO analysis results showed (Figure 6A) that the up-regulated miRNAs in the 14d-EVs group were primarily involved in biological processes such as the Notch signaling pathway and the positive regulation of cell motility and movement and were enriched in cellular components like the Golgi apparatus and ubiquitin ligase complex. Additionally, these miRNAs were associated with molecular functions such as clathrin binding, phospholipid binding, and calcium ion binding. In contrast, down-regulated miRNAs in the 14d-EVs group were mainly involved in biological processes related to the regulation of the lipid biosynthetic process, vesicle fusion, and the lipid metabolic process and were enriched in molecular functions like ion channel activity and protein binding.

The KEGG pathway enrichment bubble diagram (Figure 6B) reveals that the target genes of differentially expressed miRNAs were predominantly enriched in osteogenic differentiation-related pathways, including the MAPK signaling pathway, calcium signaling pathway, and Notch signaling pathway. These enrichment analyses indicated that the miRNAs from 14d-EVs were closely associated with osteogenic differentiation and cell migration. These findings suggest that DFATs-derived conditioned EVs might promote the osteogenic differentiation of BMSCs through miRNAs.

## 4. Discussion

Stem cells are extensively utilized in tissue engineering and regenerative medicine [26], and DFATs with stem cell properties are gradually receiving attention in this field. BMSCs, commonly used in bone tissue engineering, require invasive procedures for their extraction. Adipose tissue has the advantages of large cell reserves and easy access, positioning it as an ideal source of seed cells in tissue engineering [9]. A report indicated that DFATs could be isolated from human buccal fat pads using minimally invasive techniques, which involve only a 2 cm intraoral incision to obtain adipose tissue for DFATs cultivation [27].

Adipose tissue can be stratified into three distinct layers following digestion with collagenase and centrifugation. From top to bottom, these layers are the floating cell layer, the transparent liquid layer, and the stromal vascular fraction (SVF). Among floating cells, approximately 97% are mononuclear mature adipocytes filled with lipid droplets, while about 3% are mature adipocytes that have lost lipid droplets [28]. DFATs are isolated using the “ceiling culture” method from these floating cells, whereas ASCs are obtained from the SVF, which is a heterogeneous mixture of various cell types [29]. Consequently, ASCs are derived from a more heterogeneous cell population, whereas DFATs are more uniform in origin and exhibit a higher homogeneity. Moreover, studies have demonstrated that DFATs possess a superior proliferation capacity and osteogenic differentiation potential compared to ASCs [11,30]. However, Xue M et al. highlighted several challenges facing the application of DFATs [31]. A primary limitation is the absence of specific genetic markers to clearly define the dedifferentiation state. Additionally, the culture of DFATs lacks sufficient purity, tracking the dedifferentiation process of adipocytes in vivo remains difficult, and the fate of excreted lipid droplets is still unknown, all of which hinder progress in this field. Overall, DFATs still hold promise as highly effective seed cells in regenerative medicine.

This study is the first to investigate the potential application of EVs derived from DFATs in bone regeneration. We employed conditioned EVs from DFATs to promote the osteogenic differentiation of BMSCs, thereby circumventing the immunomodulatory disorders and potential tumor formation risks associated with stem cell therapy [32]. Previous researchers utilized DFATs combined with polylactic-co-glycolic acid (PLGA) and transplanted them into three-wall periodontal bone defects in the maxillary first molars of rats. The results confirmed that DFATs combined with PLGA could enhance bone regeneration [33]. A recent study employed cell sheet technology to compare the efficacy of DFATs and ASCs in repairing alveolar bone defects in SD rats, revealing that DFATs demonstrated a superior ability to promote bone defect repair [11]. Recent studies have identified EVs released by stem cells as crucial regulators of cell communication, with numerous investigations confirming that EVs from various stem cell types can enhance bone repair [34,35,36]. However, the role of DFATs-derived EVs in regenerative medicine remains unexplored. Given these considerations, our selection of DFATs as the source of EVs represents a novel approach.

EVs are vesicles with a phospholipid bilayer structure released by cells. They carry diverse biological elements derived from their source cells, including proteins (e.g., cytoskeletal proteins, cytokines, and growth factors), lipids (e.g., cholesterol, lipid rafts, and ceramides), and nucleic acids (e.g., DNAs, mRNAs, and miRNAs). EVs play a vital role in intercellular communication and the regulation of physiological and pathological processes. With low immunogenicity and no capacity for self-replication, EVs-based cell-free therapies can potentially minimize immune rejection and tumorigenicity [37]. Overall, EVs offer advantages such as low toxicity, low immunogenicity, high biocompatibility, and suitability for long-term storage and transport, providing new approaches for regenerative medicine.

Numerous methods are available for isolating EVs, and the choice of an appropriate technique should be guided by the research objectives and experimental conditions. Specifically, the methods for isolating EVs include ultracentrifugation, ultrafiltration, poly-ethylene glycol-based precipitation (PEG), immunoaffinity capture (IA), size-exclusion chromatography (SEC), and microfluidics (MF) [38]. Each technique has distinct advantages and limitations concerning sample purity, yield, and equipment requirements. Among these, ultrafiltration, PEG, and SEC yield higher EVs quantities, although ultrafiltration and PEG result in lower purity. For applications requiring high-purity EVs, ultracentrifugation, IA and MF are preferable [39]. SEC is based on the distribution and flow rate of different particles in the gel filtration matrix to screen molecules of different sizes, which is particularly advantageous for isolating EVs of specific sizes [40]. Ultracentrifugation, considered as the gold standard, separates particles by size and density through sedimentation under ultra-high-speed centrifugation [41]. Given its balance of yield, purity, cost, and efficiency, ultracentrifugation was selected for EVs isolation in this study.

The optimal osteogenic induction duration for EVs varies depending on the cell type. The present study demonstrated that EVs derived from DFATs with 14 days of osteogenic induction produced the highest ALP activity when applied to BMSCs at a concentration of 10 μg/mL. Other studies have also corroborated that EVs derived from stem cells with osteogenic induction exhibit a more pronounced bone-promoting effect. Zhu et al. isolated EVs from human alveolar bone-derived bone marrow mesenchymal stromal cells (AB-BMSCs), revealing that EVs secreted by AB-BMSCs after 7 days of osteogenic induction effectively promoted the osteogenic differentiation of BMSCs [22]. Liu et al. demonstrated that osteogenic induction for 3, 7, or 14 days enhanced the bone-promoting ability of PDLSCs-derived EVs, with 14 days of induction showing the greatest capacity to promote BMSCs’ osteogenic differentiation [20]. Additionally, study reported that the EVs of stem cells from human exfoliated deciduous teeth (SHEDs) with 3 days of osteogenic induction could promote PDLSCs osteogenic differentiation via the Wnt/β-catenin and BMP/Smad signaling pathways [42].

The mechanism through which conditioned EVs promote osteogenesis in BMSCs might involve changes in the miRNA expression profile. MiRNAs within EVs represent a critical class of bioactive molecules, playing a pivotal role in intercellular communication, immune modulation, and tissue repair [43]. In the present study, we compared differentially expressed miRNAs using the high-throughput sequencing of DFATs-derived EVs at days 0 and 14 of osteogenic induction. The results indicated that 44 miRNAs were significantly up-regulated and 50 miRNAs were significantly down-regulated in conditioned EVs. Notably, several differentially expressed miRNAs, including miR-378a-3p, miR-16-5p, and miR-221-3p have previously been associated with osteogenesis. A recent study demonstrated that tantalum particles enhanced the proliferation, migration, and osteogenic differentiation of BMSCs by upregulating miR-378a-3p via macrophage-derived EVs [44]. Another study established that miR-16-5p, enriched in BMSCs-derived EVs, promoted osteogenic differentiation by targeting Axin2, a negative regulator of the Wnt/β-catenin pathway [45]. Additionally, research has indicated that miR-221-3p suppresses the osteogenic differentiation of BMSCs through the IGF-1/ERK pathway, with its down-regulation potentially alleviating alveolar bone loss in diabetic patients [46]. These findings reveal that alterations in miRNA expression profiles might be the downstream mechanism responsible for the facilitation of osteogenesis by conditioned EVs.

## 5. Conclusions

In summary, the present study demonstrated that conditioned EVs derived from DFATs enhanced the proliferation, migration, and osteogenic differentiation of BMSCs, with the underlying mechanism potentially linked to differential miRNA expression in EVs. These findings highlight the potential of DFATs-based cell-free therapy in regenerative medicine and offer novel research directions and methodologies for bone tissue regeneration.

## Data Availability

The datasets used and analyzed during the current study are available from the corresponding author on reasonable request.

## References

[1] Han S., Yang H., Ni X., Deng Y., Li Z., Xing X., Du M. (2023). Programmed release of vascular endothelial growth factor and exosome from injectable chitosan nanofibrous microsphere-based PLGA-PEG-PLGA hydrogel for enhanced bone regeneration. Int. J. Biol. Macromol..

[2] Qiao X., Tang J., Dou L., Yang S., Sun Y., Mao H., Yang D. (2023). Dental Pulp Stem Cell-Derived Exosomes Regulate Anti-Inflammatory and Osteogenesis in Periodontal Ligament Stem Cells and Promote the Repair of Experimental Periodontitis in Rats. Int. J. Nanomed..

[3] Chen C., Fu L., Luo Y., Zeng W., Qi X., Wei Y., Chen L., Zhao X., Li D., Tian M. (2023). Engineered Exosome-Functionalized Extracellular Matrix-Mimicking Hydrogel for Promoting Bone Repair in Glucocorticoid-Induced Osteonecrosis of the Femoral Head. ACS Appl. Mater. Interfaces.

[4] Zhuang Y., Cheng M., Li M., Cui J., Huang J., Zhang C., Si J., Lin K., Yu H. (2022). Small extracellular vesicles derived from hypoxic mesenchymal stem cells promote vascularized bone regeneration through the miR-210-3p/EFNA3/PI3K pathway. Acta Biomater..

[5] Brennan M.Á., Layrolle P., Mooney D.J. (2020). Biomaterials functionalized with MSC secreted extracellular vesicles and soluble factors for tissue regeneration. Adv. Funct. Mater..

[6] Liu A., Lin D., Zhao H., Chen L., Cai B., Lin K., Shen S.G. (2021). Optimized BMSC-derived osteoinductive exosomes immobilized in hierarchical scaffold via lyophilization for bone repair through Bmpr2/Acvr2b competitive receptor-activated Smad pathway. Biomaterials.

[7] Álvarez-Viejo M. (2020). Mesenchymal stem cells from different sources and their derived exosomes: A pre-clinical perspective. World J. Stem Cells.

[8] Zollino I., Zuolo M., Gianesini S., Pedriali M., Sibilla M.G., Tessari M., Carinci F., Occhionorelli S., Zamboni P. (2017). Autologous adipose-derived stem cells: Basic science, technique, and rationale for application in ulcer and wound healing. Phlebology.

[9] Zhou L.N., Wang J.C., Zilundu P.L.M., Wang Y.Q., Guo W.P., Zhang S.X., Luo H., Zhou J.H., Deng R.D., Chen D.F. (2020). A comparison of the use of adipose-derived and bone marrow-derived stem cells for peripheral nerve regeneration in vitro and in vivo. Stem Cell Res. Ther..

[10] Liang Z., He Y., Tang H., Li J., Cai J., Liao Y. (2023). Dedifferentiated fat cells: Current applications and future directions in regenerative medicine. Stem Cell Res. Ther..

[11] Huang G., Xia B., Dai Z., Yang R., Chen R., Yang H. (2022). Comparative study of dedifferentiated fat cell and adipose-derived stromal cell sheets for periodontal tissue regeneration: In vivo and in vitro evidence. J. Clin. Periodontol..

[12] Akita D., Kazama T., Tsukimura N., Taniguchi Y., Takahashi R., Arai Y., Tsurumachi-Iwasaki N., Yasuda H., Okubo T., Kano K. (2022). Transplantation of Mature Adipocyte-Derived Dedifferentiated Fat Cells Facilitates Periodontal Tissue Regeneration of Class II Furcation Defects in Miniature Pigs. Materials.

[13] Takabatake K., Matsubara M., Yamachika E., Fujita Y., Arimura Y., Nakatsuji K., Nakano K., Nagatsuka H., Iida S. (2021). Comparing the Osteogenic Potential and Bone Regeneration Capacities of Dedifferentiated Fat Cells and Adipose-Derived Stem Cells In Vitro and In Vivo: Application of DFAT Cells Isolated by a Mesh Method. Int. J. Mol. Sci..

[14] Sugihara H., Yonemitsu N., Miyabara S., Yun K. (1986). Primary cultures of unilocular fat cells: Characteristics of growth in vitro and changes in differentiation properties. Differentiation.

[15] Chai Y., Chen Y., Yin B., Zhang X., Han X., Cai L., Yin N., Li F. (2022). Dedifferentiation of Human Adipocytes After Fat Transplantation. Aesthetic Surg. J..

[16] Yanagi T., Kajiya H., Fujisaki S., Maeshiba M., Yanagi-S A., Yamamoto-M N., Kakura K., Kido H., Ohno J. (2021). Three-dimensional spheroids of dedifferentiated fat cells enhance bone regeneration. Regen. Ther..

[17] Fujii S., Endo K., Matsuta S., Komori K., Sekiya I. (2022). 2022. Comparison of the yields and properties of dedifferentiated fat cells and mesenchymal stem cells derived from infrapatellar fat pads. Regen. Ther..

[18] Jumabay M., Zhang L., Yao J., Boström K.I. (2022). Progenitor cells from brown adipose tissue undergo neurogenic differentiation. Sci. Rep..

[19] Shimizu M., Matsumoto T., Kikuta S., Ohtaki M., Kano K., Taniguchi H., Saito S., Nagaoka M., Tokuhashi Y. (2018). Transplantation of dedifferentiated fat cell-derived micromass pellets contributed to cartilage repair in the rat osteochondral defect model. J. Orthop. Sci..

[20] Liu T., Hu W., Zou X., Xu J., He S., Chang L., Li X., Yin Y., Tian M., Li Z. (2020). Human Periodontal Ligament Stem Cell-Derived Exosomes Promote Bone Regeneration by Altering MicroRNA Profiles. Stem Cells Int..

[21] Zhu W., Wang Q., Zhang J., Sun L., Hong X., Du W., Duan R., Jiang J., Ji Y., Wang H. (2023). Exosomes derived from mir-214-3p overexpressing mesenchymal stem cells promote myocardial repair. Biomater. Res..

[22] Zhu Q., Tang Y., Zhou T., Yang L., Zhang G., Meng Y., Zhang H., Gao J., Wang C., Su Y.X. (2023). Exosomes derived from mesenchymal stromal cells promote bone regeneration by delivering miR-182-5p-inhibitor. Pharmacol. Res..

[23] Xu T., Luo Y., Wang J., Zhang N., Gu C., Li L., Qian D., Cai W., Fan J., Yin G. (2020). 2020. Exosomal miRNA-128-3p from mesenchymal stem cells of aged rats regulates osteogenesis and bone fracture healing by targeting Smad5. J. Nanobiotechnol..

[24] Liu L., Guo S., Shi W., Liu Q., Huo F., Wu Y., Tian W. (2021). Bone Marrow Mesenchymal Stem Cell-Derived Small Extracellular Vesicles Promote Periodontal Regeneration. Tissue Eng. Part A.

[25] Görgens A., Corso G., Hagey D.W., Wiklander J., Gustafsson M.O., Felldin U., Lee Y., Bostancioglu R.B., Sork H., Liang X. (2022). Identification of storage conditions stabilizing extracellular vesicles preparations. J. Extracell. Vesicles.

[26] Matsuzaka Y., Yashiro R. (2024). Current Strategies and Therapeutic Applications of Mesenchymal Stem Cell-Based Drug Delivery. Pharmaceuticals.

[27] Kishimoto N., Momota Y., Hashimoto Y., Tatsumi S., Ando K., Omasa T., Kotani J. (2014). The osteoblastic differentiation ability of human dedifferentiated fat cells is higher than that of adipose stem cells from the buccal fat pad. Clin. Oral Investig..

[28] Sakuma T., Matsumoto T., Kano K., Fukuda N., Obinata D., Yamaguchi K., Yoshida T., Takahashi S., Mugishima H. (2009). Mature, adipocyte derived, dedifferentiated fat cells can differentiate into smooth muscle-like cells and contribute to bladder tissue regeneration. J. Urol..

[29] Labusca L. (2022). Adipose tissue in bone regeneration—Stem cell source and beyond. World J. Stem Cells.

[30] Watson J.E., Patel N.A., Carter G., Moor A., Patel R., Ghansah T., Mathur A., Murr M.M., Bickford P., Gould L.J. (2014). Comparison of Markers and Functional Attributes of Human Adipose-Derived Stem Cells and Dedifferentiated Adipocyte Cells from Subcutaneous Fat of an Obese Diabetic Donor. Adv. Wound Care.

[31] Xue M., Liao Y., Jiang W. (2024). Insights into the molecular changes of adipocyte dedifferentiation and its future research opportunities. J. Lipid Res..

[32] Wang D., Cao H., Hua W., Gao L., Yuan Y., Zhou X., Zeng Z. (2022). Mesenchymal Stem Cell-Derived Extracellular Vesicles for Bone Defect Repair. Membranes.

[33] Suzuki D., Akita D., Tsurumachi N., Kano K., Yamanaka K., Kaneko T., Kawano E., Iguchi S., Toriumi T., Arai Y. (2017). Transplantation of mature adipocyte-derived dedifferentiated fat cells into three-wall defects in the rat periodontium induces tissue regeneration. J. Oral Sci..

[34] Sun T., Feng Z., He W., Li C., Han S., Li Z., Guo R. (2023). Novel 3D-printing bilayer GelMA-based hydrogel containing BP,β-TCP and exosomes for cartilage-bone integrated repair. Biofabrication.

[35] Lei F., Li M., Lin T., Zhou H., Wang F., Su X. (2022). Treatment of inflammatory bone loss in periodontitis by stem cell-derived exosomes. Acta Biomater..

[36] Zhao Y., Gong Y., Liu X., He J., Zheng B., Liu Y. (2022). The Experimental Study of Periodontal Ligament Stem Cells Derived Exosomes with Hydrogel Accelerating Bone Regeneration on Alveolar Bone Defect. Pharmaceutics.

[37] Moghassemi S., Dadashzadeh A., Sousa M.J., Vlieghe H., Yang J., León-Félix C.M., Amorim C.A. (2024). Amorim. Extracellular vesicles in nanomedicine and regenerative medicine: A review over the last decade. Bioact. Mater..

[38] Tosar J.P., Useckaite Z., Valle F., Varga Z., van der Pol E., van Herwijnen M.J.C., Wauben M.H.M., Wehman A.M., Williams S., Zendrini A. (2024). Witwer. Minimal information for studies of extracellular vesicles (MISEV2023): From basic to advanced approaches. J. Extracell. Vesicles.

[39] Sidhom K., Obi P.O., Saleem A. (2020). A Review of Exosomal Isolation Methods: Is Size Exclusion Chromatography the Best Option?. Int. J. Mol. Sci..

[40] Monguió-Tortajada M., Gálvez-Montón C., Bayes-Genis A., Roura S., Borràs F.E. (2019). Extracellular vesicle isolation methods: Rising impact of size-exclusion chromatography. Cell. Mol. Life Sci..

[41] Altıntaş Ö., Saylan Y. (2023). Exploring the Versatility of Exosomes: A Review on Isolation, Characterization, Detection Methods, and Diverse Applications. Anal. Chem..

[42] Wang M., Li J., Ye Y., He S., Song J. (2020). SHED-derived conditioned exosomes enhance the osteogenic differentiation of PDLSCs via Wnt and BMP signaling in vitro. Differentiation.

[43] Li B., Cao Y., Sun M., Feng H. (2021). Expression, regulation, and function of exosome-derived miRNAs in cancer progression and therapy. FASEB J..

[44] Yang J., Gong X., Li T., Xia Z., He R., Song X., Wang X., Wu J., Chen J., Wang F. (2024). Tantalum Particles Promote M2 Macrophage Polarization and Regulate Local Bone Metabolism via Macrophage-Derived Exosomes Influencing the Fates of BMSCs. Adv. Healthc. Mater..

[45] Duan J., Li H., Wang C., Yao J., Jin Y., Zhao J., Zhang Y., Liu M., Sun H. (2023). BMSC-derived extracellular vesicles promoted osteogenesis via Axin2 inhibition by delivering MiR-16-5p. Int. Immunopharmacol..

[46] Gan K., Dong G.H., Wang N., Zhu J.F. (2020). miR-221-3p and miR-222-3p downregulation promoted osteogenic differentiation of bone marrow mesenchyme stem cells through IGF-1/ERK pathway under high glucose condition. Diabetes Res. Clin. Pract..

